# Correspondence: Reply to ‘Strongly-driven Re+CO_2_ redox reaction at high-pressure and high-temperature'

**DOI:** 10.1038/ncomms13538

**Published:** 2016-11-29

**Authors:** Mario Santoro, Federico A. Gorelli, Roberto Bini, Ashkan Salamat, Gaston Garbarino, Claire Levelut, Olivier Cambon, Julien Haines

**Affiliations:** 1 Istituto Nazionale di Ottica, Consiglio Nazionale delle Ricerche (INO-CNR), 50019 Sesto Fiorentino, Italy; 2 European Laboratory for Non Linear Spectroscopy (LENS), 50019 Sesto Fiorentino, Italy; 3 Dipartimento di Chimica dell'Università di Firenze, 50019 Sesto Fiorentino, Italy; 4 European Synchrotron Radiation Facility, 38343 Grenoble, France; 5 Laboratoire Charles Coulomb, UMR 5221, Centre National de la Recherche Scientifique (CNRS), Département Colloïdes, Verres et Nanomatériaux (CVN), Université Montpellier 2, 34095 Montpellier, France; 6 Institut Charles Gerhardt Montpellier, UMR 5253, Centre National de la Recherche Scientifique (CNRS), Equipe C2M, Université Montpellier 2, 34095 Montpellier, France

Santamaria-Perez *et al*.^1^ attempt to reproduce our CO_2_–SiO_2_ solid solution^2^. In their study^1^, mixtures of CO_2_ and SiO_2_ similar to those studied in our work^2^ were indirectly laser heated up to 2,400 K and 50 GPa by using Re as an internal heater, and a CO_2_–SiO_2_ solid solution was not obtained. Instead, the only temperature quenched crystalline phases identified by X-ray diffraction were: known polymorphs of pure CO_2_ and SiO_2_, Re and ReO_2_. In particular, the structure of ReO_2_ was identified as being the β-ReO_2_ (Pbcn), which is a well known phase of this oxide discovered many decades ago (ref. 9 in the letter). ReO_2_ was then inferred to form from a high P–T, Re+CO_2_ redox reaction. Moreover, it is shown that β-ReO_2_ may provide a better fit to our XRD pattern than the cristobalite-like CO_2_–SiO_2_ solid solution. A shadow is then cast on the very existence of this solid solution.

In light of this study^1^, we have reanalysed all our data, and realized that more complex chemical reactions may have occurred in our samples due to the extreme high temperatures: T>4,000 K. To make the reaction between CO_2_ and SiO_2_ as efficient as possible, we understood that starting from confined CO_2_ in a SiO_2_ zeolite was not sufficient and then we planned and managed to heat the sample at conditions where both CO_2_ and SiO_2_ are fluid. The aim was to react them together starting from an ideal, hot, highly mobile mixture. For achieving very high temperatures, we insulated diamond using thick NaCl layers pelleted on it. Then, in principle, we carefully avoided laser heating the Re gasket. Nevertheless, we recognize that the laser spot may have hit the gasket at some point and/or the hot fluid sample may have drifted toward the gasket where spurious reactions with Re may have occurred. We have now reanalysed all our XRD patterns, including those at room pressure where potential volatile components (for example, CO_2_) are absent making data interpretation as simple and clean as possible. Indeed, we confirm that the β-ReO_2_ provides a better fit to the new phase than cristobalite. The lack of XRD Bragg peaks of stishovite in our samples, which was found in the letter by Santamaria-Perez, may be easily explained with liquid SiO_2_ being temperature quenched in a glassy form. Our results thus do not prove the existence of a CO_2_–SiO_2_ solid solution and β-ReO_2_ is indeed one of the materials synthesized in our experiment. Also, Raman data show that the chemistry was even more complex in our study. We now have an alternative explanation for the dominant Raman peak with five, fine-structure components^2^. This spectrum matches exactly that of crystalline Cl_2_ (ref. 3), which clearly came from a partial dissociation of the NaCl insulating layers. Incidentally, we note that very recent studies show that simple salts, such as NaCl and KCl, may undergo major chemical changes upon laser heating at high pressures^4^,^5^ (see also ref. 5 for an updated Raman spectrum of solid Cl_2_). We recall, as noted in our original paper^2^, that some microcrystalline/amorphous carbon was also formed in our laser-heated sample. In conclusion, we think that our revised data interpretation is consistent with the very complex chemistry that occurred in our study under extreme conditions, one potential chemical reaction path being CO_2_ decomposed and, as a result, both Re from the gasket and NaCl were partially oxidized by the available free oxygen. Pure, molecular chlorine was then released after oxidization of NaCl. Decomposition of CO_2_ and oxidation of Re and NaCl finally led to free carbon available to form graphite. We can resume this potential reaction as:


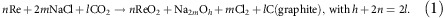


## Additional information

**How to cite this article:** Santoro, M. *et al*. Correspondence: Reply to ‘Strongly-driven Re+CO_2_ redox reaction at high-pressure and high-temperature'. *Nat. Commun.* **7,** 13538 doi: 10.1038/ncomms13538 (2016).
